# A microRNA-mediated decrease in eukaryotic initiation factor 2α promotes cell survival during PS-341 treatment

**DOI:** 10.1038/srep21565

**Published:** 2016-02-22

**Authors:** Lili Jiang, Dan Zang, Songgang Yi, Xiaofen Li, Changshan Yang, Xiaoxian Dong, Chong Zhao, Xiaoying Lan, Xin Chen, Shouting Liu, Ningning Liu, Hongbiao Huang, Xianping Shi, Xuejun Wang, Jinbao Liu

**Affiliations:** 1 State Key Lab of Respiratory Disease, Protein Modification and Degradation Lab, Department of Pathophysiology, Guangzhou Medical University, Guangdong 510182, People’s Republic of China; 2 Guangzhou Research Institute of Cardiovascular Disease, the Second Affiliated Hospital, Guangzhou Medical University, Guangzhou, Guangdong 510260, People’s Republic of China; 3 Division of Basic Biomedical Sciences, Sanford School of Medicine of the University of South Dakota, Vermillion, South Dakota 57069, USA

## Abstract

MicroRNAs (miRs) play pivotal roles in carcinogenesis and endoplasmic reticulum (ER) that performs the folding, modification and trafficking of proteins targeted to the secretory pathway. Cancer cells often endure ER stress during tumor progression but use the adaptive ER stress response to gain survival advantage. Here we report: (i) A group of miRs, including miR-30b-5p and miR-30c-5p, are upregulated by proteasome inhibitor PS-341 treatment, in HepG2 and MDA-MB-453 cells. (ii) Two representative PS-341-induced miRs: miR-30b-5p and miR-30c-5p are found to promote cell proliferation and anti-apoptosis in both tumor cells. (iii) eIF2α is confirmed as the congenerous target of miR-30b-5p and miR-30c-5p, essential to the anti-apoptotic function of these miRs. (iv) Upregulation of miR-30b-5p or miR-30c-5p, which occurs latter than the increase of phosphorylated eIF2α (p-eIF2α) in the cell under ER stress, suppresses the p-eIF2α/ATF4/CHOP pro-apoptotic pathway. (v) Inhibition of the miR-30b-5p or miR-30c-5p sensitizes the cancer cells to the cytotoxicity of proteasome inhibition. In conclusion, we unravels a new miRs-based mechanism that helps maintain intracellular proteostasis and promote cell survival during ER stress through upregulation of miR-30b-5p and miR-30c-5p which target eIF2α and thereby inhibit the p-eIF2α/ATF4/CHOP pro-apoptotic pathway, identifying miR-30b-5p and miR-30c-5p as potentially new targets for anti-cancer therapies.

MicroRNAs (miRs) are a class of endogenous non-coding small RNAs of 20–22 nucleotides and have been identified as a new type of gene expression regulators, which negatively regulate gene expression at the post-transcriptional level primarily by targeting the 3′ untranslated region (3′-UTR) of mRNAs in a sequence-specific manner^1^. Increasing numbers of reports show that miRs are involved in multiple biological processes, including cell proliferation, apoptosis, and stress response^2^,^3^. miRs are found to have key roles in the progression of various human cancers, where some miRs are dysregulated in multiple tumors and identified as onco-miRs or tumor suppressors^4^,^5^,^6^. miRs could target various mRNAs to operate extremely intricate regulatory networks and regulate the expression of genes in many pathways which are correlated with tumor initiation, development and progression^7^,^8^,^9^. Considering the close connection between miRs and carcinogenesis, miRs have been considered as potential targets for cancer diagnosis and therapy^10^,^11^,^12^.

Synthesis and maturation of all secretory proteins occur in the endoplasmic reticulum (ER)^13^. The ER performs the folding, modification and trafficking of these proteins and ER disturbance is also involved in apoptosis^13^,^14^,^15^. ER stress (ERS) occurs when the protein load exceeds the ER capacity to fold or degrade them, and is manifested by the accumulation of misfolded proteins in the ER^13^,^16^. Disturbance of protein biogenesis within the ER evokes a stress response termed the unfolded protein response (UPR) to coordinate adaptive response to counter the accumulation of misfolded proteins in the ER^17^,^18^. ER stress sensors, including inositol requiring 1 (IRE1), activated transcription factor 6 (ATF6), and ER-resident PKR-like eIF2α kinase (PERK) detect the accumulation of unfolded or misfolded proteins and trigger multiple signaling pathways aiming to alleviate ER stress^19^,^20^,^21^,^22^. The first-line of reactions to deal with ER stress is to suppress general protein synthesis via PERK-mediated eIF2α phosphorylation and IRE1-mediated cleavage of mRNAs while all the three arms of UPR signaling activate transcription cascades to synthesize selective sets of proteins that can promote protein folding and augment the degradation of unfolded/misfolded ER proteins^23^,^24^. If this response fails, mitogen-activated protein kinases (MAPKs), Jun N-terminal kinase (JNK), and nuclear factor-κB (NF-κB) might be activated to induce gene expression performing host protection^25^,^26^,^27^,^28^. Since the UPR is also characterized by the transcriptional induction of the pro-apoptotic transcription factors, the cell undergoes apoptosis if both responses fail to defend against ER stress^29^,^30^. The apoptosis induced by sustained ER stress is mediated *via* the PERK/eIF2α/ATF4 and the IRE1 signaling pathways^22^,^29^,^30^. In the former, PERK phosphorylates eIF2α and the resultant increase in p-eIF2α suppresses general translation but allows a selective set of proteins such as ATF4 to be translated; ATF4 in turn increases the transcription of CHOP and thereby activates a pro-apoptotic gene program^31^. Cancer progression with rapid proliferation of cancer cells requires augmented protein synthesis. Cancer cells usually encounter wretched microenvironmental conditions, such as nutrient deprivation, hypoxia, impaired glycosylation, and acidosis, which are known triggers of ER stress^32^. ER chaperones, such as glucose-regulated protein 78 (GRP78), glucose-regulated protein 94 (GRP94) and protein disulfide isomerase (PDI), perform essential roles in maintaining ER homeostasis, contributing to cancer cell survival and growth^32^,^33^,^34^. It has been reported that the PERK-peIF2α-ATF4 signaling pathway is important for tumor cell proliferation and progression^35^. Accumulating reports suggest a correlation between the ER stress response and tumor progression. In addition, proteasome inhibitors, such as PS-341, which have been considered as potential chemotherapeutic drugs, are reported to induce tumor cell apoptosis through induction of ER stress^36^,^37^,^38^,^39^. However, our understanding of the regulatory mechanism of the UPR triggered by ER stress remains incomplete.

Altered expressions of many miRs were associated with the UPR triggered by ER stress, serving as UPR modulators or effectors^40^,^41^; however, none of the miRs that were previously associated with ER stress or the UPR has been demonstrated to regulate the regulatory components of general protein translation or ER protein influx. In the current study, we have discovered that a new group of miRs, including miR-30b-5p, miR-30c-5p, miR-664-3p, miR-106–5p, miR-182–5p and miR-193–3p, are upregulated in HepG2 and MDA-MB-453 cancer cells after treatment with PS-341. Our further investigation demonstrates that miR-30b-5p and miR-30c-5p, which are upregulated after eIF2α phosphorylation has increased, target eIF2α, lead to suppression of the p-eIF2α-ATF4-CHOP pro-apoptotic pathway, and thereby promote cell proliferation and confer resistance to apoptosis in the cancer cells both under basal culture condition and during proteasome inhibition, unraveling a new miRs-based mechanism for suppressing general protein synthesis and improving cell survival in the UPR. Moreover, inhibition of either miRs remarkably enhanced the cytotoxicity of proteasome inhibition, which identifies these PS341-induced miRs as potential new targets for anti-cancer therapies.

## Results

### PS-341 induces upregulation of a new group of miRs

**Figure 1:**
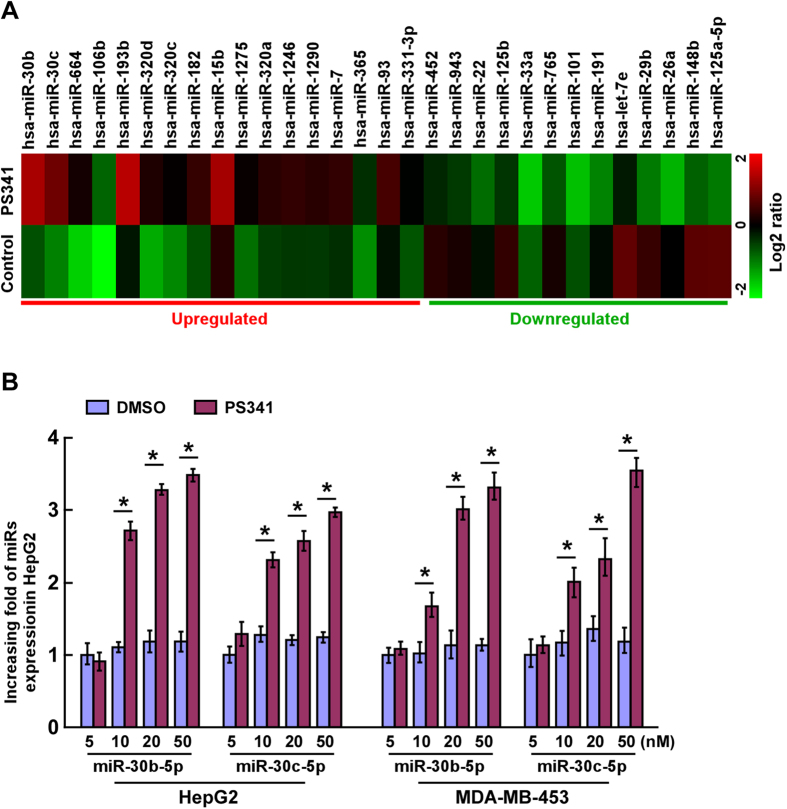
A group of miRs are upregulated in HepG2 cancer cell by PS-341 treatment. **(A)** The expression of miRs selected by miRNA-array analysis, in HepG2 cells treated with PS-341(50 nM) for 24 h, confirmed by qRT-PCR. The data analysis and visualization were performed with MeV 4.4 software (MultiExperiment Viewer; http://www.tm4.org/mev/). The pseudocolors represent the intensity scale of miRs, generated by fold-change of miR expression. (**B)** The expression of miR-30b-5p and miR-30c-5p in HepG2 and MDA-MB-453 cells treated with indicated concentrations (0, 10, 20, 50 nM) of PS-341 for 24 h, determined with qRT-PCR. Transcript levels were normalized using U6 expression. Each bar represents the mean ± SD of three independent experiments. *P < 0.05 *vs*. respective vehicle control treatment.

A microarray analysis for miR gene expression was performed in hepatocellular carcinoma HepG2 cells after treatment with a proteasome inhibitor PS-341 (50 nM) for 24 h. A variety of miRs, including miR-30b-5p, miR-30c-5p, miR-664–3p, miR-106–5p, miR-182-5p and miR-193–3p, were found to be upregulated, and the upregulation of miR-30b-5p and miR-30c-5p was found to be the most significant (Fig. 1A). To confirm the results of microarray analysis, the expressions of miR-30b-5p and miR-30c-5p were further examined in a proteasome inhibitor PS-341 dose-dependent manner, by real-time PCR analysis (qPCR) in both HepG2 and MDA-MB-453 cells (Fig. 1B). The expression of these miRs was all upregulated dose-dependently in cells treated with PS-341, compared with the control cells. Taken together, these data indicate that the expressions of miR-30b-5p and miR-30c-5p can be upregulated by PS-341 treatment.

### PS-341-induced miR-30b-5p and miR-30c-5p promote tumor cell viability and anti-apoptosis function

**Figure 2:**
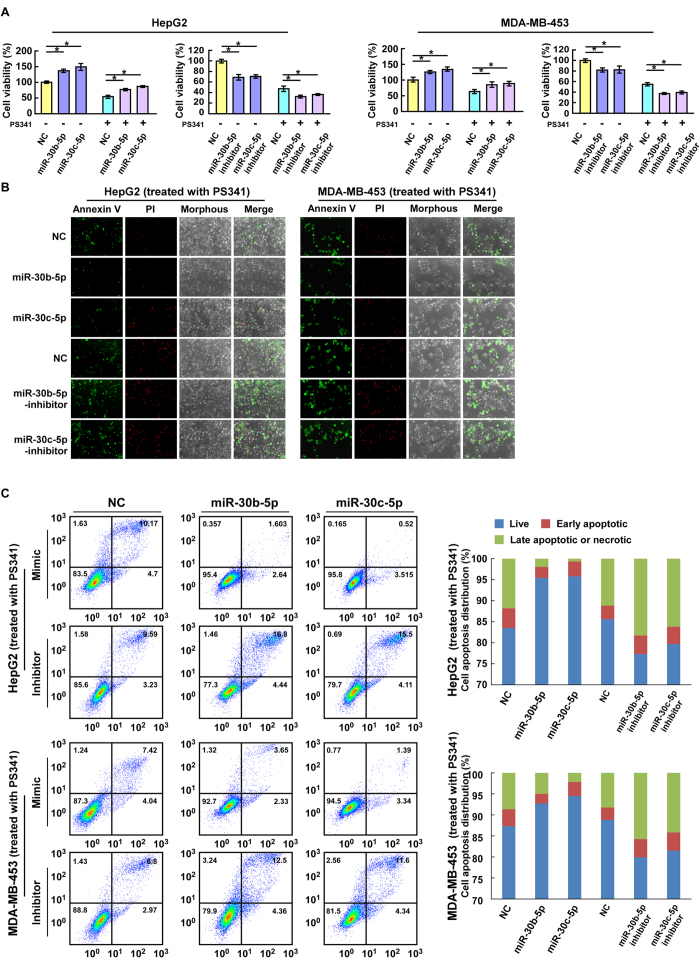
The miR-30b-5p and miR-30c-5p upregulated by PS-341 promote tumor cell viability and anti-apoptosis function. **(A)** Effects of ectopic miR-30b-5p and miR-30c-5p mimics or inhibitors on the cell viability, analyzed by the CCK8 assay. Each bar represents the mean ± SD of three independent experiments. NC, non-specific control; *P < 0.05. **(B)** The cytotoxicity detected with Annxin V/PI immunofluorescent staining. The cells were treated with PS-341 at concentration 50 nM for 24 h. **(C)** Apoptosis analysis by flow cytometry after treatment with PS-341.

To begin to elucidate the biological significance of these PS-341-induced miRs, we performed a variety of cell biology experiments to explore their role in tumor progression. The CCK8 assay showed that cell viability was increased by ectopic expression of miR-30b-5p or miR-30c-5p in both HepG2 and MDA-MB-453 cells; and conversely, the cell viability was decreased by inhibition of miR-30b-5p or miR-30c-5p with specific inhibitor both under the basal cell culture condition and after PS-341 treatment (50 nM, 24 h) (Fig. 2A). Moreover, the cytotoxicity was assessed using Annxin V immunofluorescent staining coupled with propidium iodide (PI) staining. After treatment with PS-341, the percentage of apoptotic and necrotic cells (FITC Annxin V^+^/PI^+^) was significantly lower in HepG2 and MDA-MB-453 cells overexpressing either of the two PS341-induced miRs, and conversely, was higher in cells with either one of these miRs being inhibited with specific inhibitor, compared with negative control cells (Fig. 2B). These results have also been quantified by flow cytometry. Consistent with fluorescence microscopic analysis, apoptosis analysis by flow cytometry showed that after treatment with PS-341, the percentage of apoptotic and necrotic cells decreased in HepG2 and MDA-MB-453 cells overexpressing either of these miRs, while increased in cells with inhibition of either of these miRs (Fig. 2C). These results show that the PS341-induced miRs enhance cell viability and attenuate PS-341-induced apoptosis in both HepG2 and MDA-MB-453 cells, suggesting an essential function of these miRs in PS341-induced adaptive ER stress responses.

### PS-341-induced miR-30b-5p and miR-30c-5p regulate ER stress signaling pathway *via* targeting eIF2α

**Figure 3:**
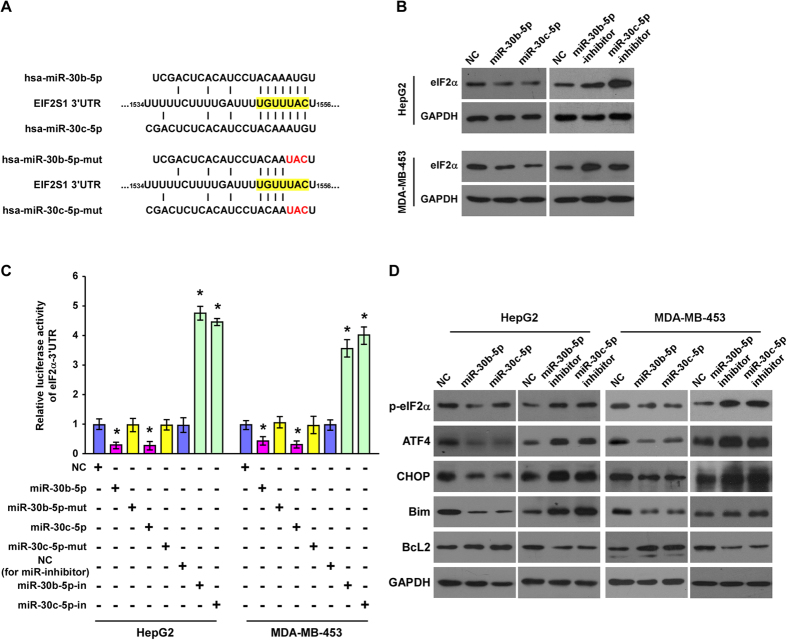
miR-30b-5p and miR-30c-5p target eIF2α and suppress ATF4 signaling. **(A)** Schematic representation of the target sites of miR-30b-5p and miR-30c-5p in the 3′-UTR of *eIF2α* mRNA. (**B**) Western blot analyses for eIF2α protein in the indicated cells treated with miR-30b-5p and miR-30c-5p mimics or inhibitors. GAPDH was the loading control. **(C)** Luciferase assays of indicated cells transfected with the pGL3- eIF2α-3′UTR reporter and the mimic, mutant, or inhibitors of miR-30b-5p and miR-30c-5p. (**D**) Western blot analyses for ER stress response related proteins in the indicated cells treated with the mimic or inhibitor of miR-30b-5p and miR-30c-5p. GAPDH served as the loading control. Each bar represents the mean ± SD of three independent experiments. *P < 0.05 *vs*. the respective NC.

Since miR-30b-5p and miR-30c-5p are upregulated by PS-341 and are pro-survival and anti-apoptotic in the cancer cells, and PS-341-induced protein accumulation leads to an ER stress^36^,^37^,^38^,^39^, we then examined the potential interaction between these miRs and the UPR pathways. The publicly available algorithm (TargetScan 6.2) was used to map the potential targets of miR-30b-5p and miR-30c-5p. We found that eIF2α (*EIF2S1*, NM_004094) was the shared target of both miRs (Fig. 3A). As predicted, western blot analysis showed that eIF2α protein level was downregulated by ectopic expression of either miR, and conversely, upregulated by their inhibitors in both HepG2 and MDA-MB-453 cells (Fig. 3B). To further confirm whether eIF2α is a direct target of these two miRs, the pGL3- eIF2α-3′-UTR-luciferase reporter was constructed, which contains all the putative miR binding sites. The luciferase reporter assay showed that ectopic expression of either miR-30b-5p or miR-30c-5p could decrease the luciferase activity of the reporter, and conversely, suppression of either of these miRs significantly increased the reporter activity (Fig. 3C). By contrast, the mutant form of these miRs, each containing three altered bases in the seed sequence (Fig. 3A), failed to show an inhibitory effect on the reporter luciferase activity (Fig. 3C). These results confirm that miR-30b-5p and miR-30c-5p can specifically target eIF2a in both HepG2 and MDA-MB-453 cells.

It has been reported that eIF2α is one of the essential regulators in the UPR signaling pathway. We then examined the expression of ER stress related proteins downstream of the PERK-eIF2α branch of the UPR. As shown in Fig. 3D, the expression of p-eIF2α, ATF4, CHOP and Bim were substantially downregulated by ectopic expression of miR-30b-5p or miR-30c-5p, and conversely was upregulated by specific inhibition of either these two miRs. BcL-2, an essential anti-apoptosis factor, was found to be upregulated in cells overexpressing miR-30b-5p or miR-30c-5p and downregulated in cells inhibiting miR-30b-5p or miR-30c-5p. Collectively, these results suggest that eIF2α is the direct target gene of miR-30b-5p and miR-30c-5p, and that these two miRs can suppress the ATF4-CHOP signaling pathway *via* targeting and suppressing eIF2α.

**Figure 4:**
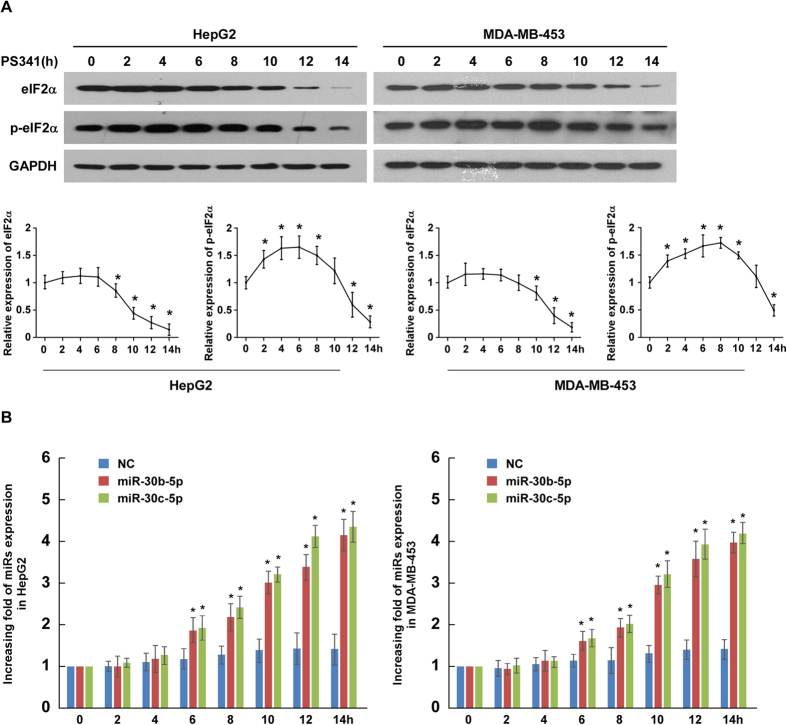
A time course comparison among eIF2α down-regulation, eIF2α phosphorylation, and changes in miR-30b-5p and miR-30c-5p expression in the cells after initiation of proteasome inhibition. HepG2 and MDA-MB-453 cells were harvested at the indicated time points after the initiation of treatment with PS341 (50 nM), for western blot analyses of the indicated proteins (**A**) and qRT-PCR analyses of the indicated miRs (**B**). (**A**) Western blot analyses of eIF2α and p-eIF2α in the indicated cells. Lower panel, quantification of western blot analyses by the Quantity One software. GAPDH was the loading control. (**B**) qRT-PCR analyses of miR-30b-5p and miR-30c-5p in the indicated cells. Transcript levels were normalized using U6 expression. Each bar represents the mean ± SD of three independent experiments; *P < 0.05 *vs*. the NC of the same time point.

To further delineate the functional relationship between UPR activation and the induction of miR-30b-5p and miR-30c-5p, we compared the time courses of changes in the protein levels of native eIF2α and p-eIF2α with the time course of these two miRs upregulation in both HepG2 and MDA-MB-453 cells treated with proteasome inhibitor PS341 (50 nM). As early as 2 h after the initiation of PS341 treatment, p-eIF2α showed significant increases, peaked at 4 h, remained high at 6 h and 8h, and returned to the baseline level at 10 h, and thereafter dropped to a level below the baseline in HepG2 cells; a similar time course of p-eIF2α increases was observed in MDA-MB-453 cells (Fig. 4A), indicating that ER stress and the UPR are triggered as early as 2 h after PS341 treatment. As shown by Fig. 4B, the upregulation of the miRs did not occur until 6 h after PS41 treatment, suggesting that the upregulation of miR-30b-5p and miR-30c-5p follows ER stress and might be an integral part, or a result, of the UPR. Equally importantly, the native form of eIF2α did not show significant decreases until 2 or more hours after the increases of the miRs become discernible in HepG2 or MDA-MB-453 cells, respectively (Fig. 4). This sequela further corroborates that these PS-341-induced miRs target eIF2α, as shown in Fig. 3. Meanwhile, eIF2α expression was maintained by miR-30b-5p or miR-30c-5p inhibitor in both HepG2 and MDA-MB-453 cells treated with PS341 (Supplementary Fig. 1). These results also suggest that these miRs might be upregulated by PS-341-induced ER stress but do not come into play until increased p-eIF2α begins to fade away.

### Downregulation of eIF2α is essential for the PS-341-induced miRs to promote cell survival

**Figure 5:**
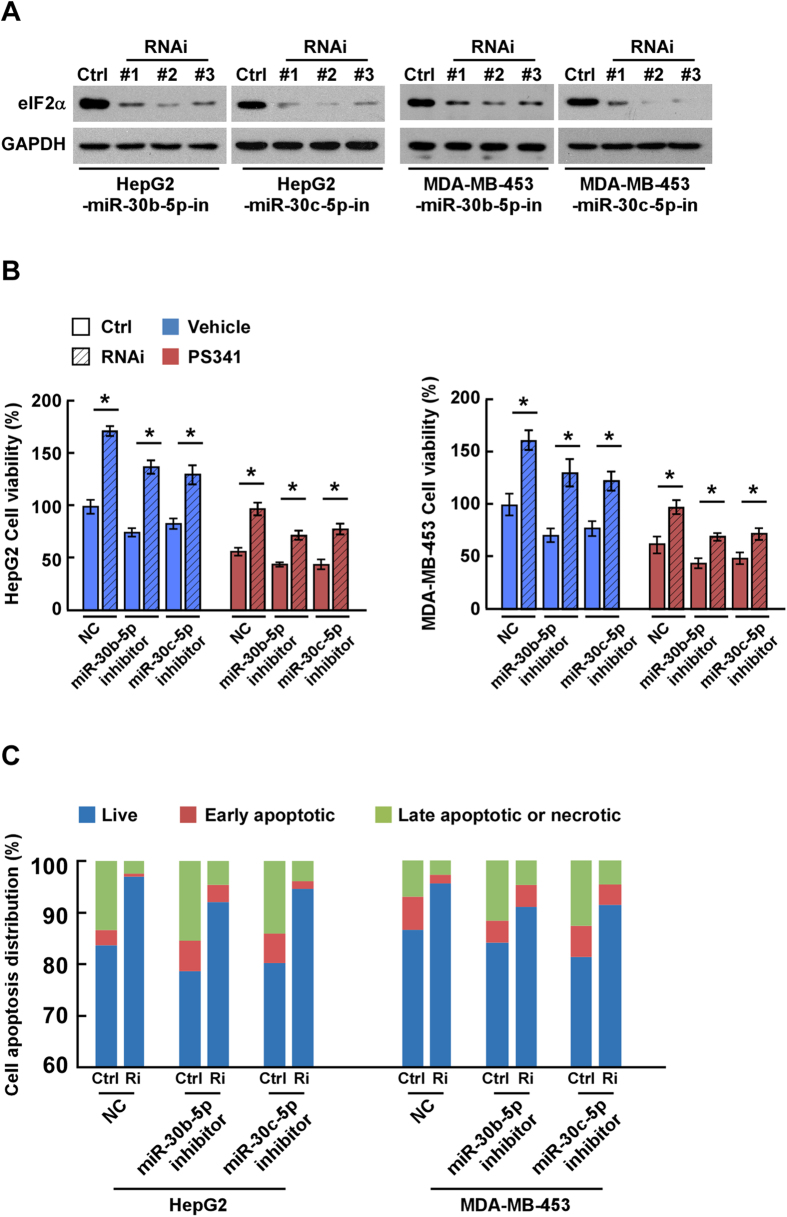
Downregulation of eIF2α is essential for the pro-survival and anti-apoptotic action of miR-30b-5p and miR-30c-5p. **(A)** Western blot analyses for eIF2α in the indicated cells that were transfected with three different siRNA specific to eIF2α (siRNA #1, #2, #3). GAPDH served as the loading control. **(B)** The cells viability of the indicated cells analyzed by the CCK8 assay. Each bar represents the mean ± SD of three independent experiments; *P < 0.05 *vs*. the NC of the same time point. **(C)** Flow cytometry analysis of cell death of the indicated cells. Ctrl, siR-Ribo™ Negative Control for siRNA; Vehicle, DMSO control; NC, micrOFF® miR inhibitor Negative Control.

To determine the role of eIF2α downregulation in the anti-apoptosis action of the PS-341-induced miRs, we used three eIF2α-specific siRNAs to suppress endogenous eIF2α expression in HepG2 and MDA-MB-453 cells. The siRNA-mediated of eIF2α knockdown was confirmed by western blot analyses (Fig. 5A). The results of the CCK8 assays revealed that the proliferative ability/viability of the cells was remarkably enhanced by silencing eIF2α in both HepG2 and MDA-MB-453 cells treated with non-specific miR control (Fig. 5B). Furthermore, the apoptosis-inducing function of miR-inhibitors specific for miR-30b-5p and miR-30c-5p was completely blocked when eIF2α was silenced (Fig. 5B). We also found that silencing eIF2α led to inhibition of PS-341-induced apoptosis, resulting in a higher level of cell viability; and the effect of the specific miR-inhibitors was also blocked by eIF2α silence in cells treated with PS-341(Fig. 5B). These results were further corroborated by flow cytometry analyses of cell death. As shown in Fig. 5C, the percentage of apoptotic and necrotic cells with PS-341 treatment was much lower in HepG2 and MDA-MB-453 cells with siRNA-mediated eIF2α silence than those without; and consistently the pro-apoptotic effects of the inhibition of these PS-341-induced miRs in PS-341-treated cells were also abolished by eIF2α knockdown (Fig. 5C).Concomitant overexpression of an eIF2α (eIF2α-ORF) that is resistant to miR-30b-5p and miR-30c-5p in both HepG2 and MDA-MB-453 cells robustly abrogated the anti-apoptotic effect of overexpression of miR-30b-5p or miR-30c-5p (Supplementary Fig. 2). Taken together, these results demonstrate that miR-30b-5p and miR-30c-5p promote cell viability/proliferation and to inhibit PS-341-induced cytotoxicity through depletion of eIF2α, in at least HepG2 and MDA-MB-453 cells.

## Discussion

The UPR triggered by overload of misfolded protein in the ER is a stress response program that reprograms cellular protein translation and gene expression to deal with proteotoxic stress in the ER. One of the primary means by which the UPR alleviates this stress is to reduce protein flux into the ER via a general suppression of protein synthesis and ER-specific mRNA degradation. It is well established that PERK-mediated phosphorylation of eIF2α plays a major role in suppression of general protein synthesis in mammalian cells during the UPR. Increased p-eIF2α also increases translation of ATF4, which drives the expression of CHOP and thereby results in activation of a pro-apoptotic gene program, leading to increase of Bim and decrease of BcL-2^31^. Here we report an additional ER stress-induced mechanism for the reduction of general protein synthesis, where PS-341-induced ER stress upregulates expression of a group of miRs after the increased phosphorylation of eIF2α has become discernible or even reached to the peak level, the PS-341-induced miRs (i.e., miR-30b-5p and miR-30c-5p) target eIF2α and decrease its protein level, resulting in decreased protein synthesis to help achieve or maintain proteostasis; meanwhile, by targeting eIF2α, these PS-341-induced miRs decrease p-eIF2α levels and thereby down-regulate ATF4 and CHOP, delaying apoptosis.

Altering miR expression by ER stress and the participation of many miRs in the ER stress response as UPR modulators or effectors in mammalian cells have been reported recently. For example, upregulation of the miR-23a ~ 27a ~ 24–2 cluster in HEK293T cells caused induction of ATF4 and CHOP and subsequent cell death^42^. miR-122 is highly expressed in normal hepatocytes but is silent or remarkably down-regulated in hepatocellular carcinoma (HCC) cells^43^. Yang *et al.*^44^ reported that overexpression of miR-122 in HCC cells led to suppression of UPR signaling via a CDK4-PSMD10 pathway and sensitized to cisplatin-triggered apoptosis. Duan and colleague^45^ showed that ER stress downregulated the expression of the miR-199a/miR-214 cluster in HCC cells and further demonstrated that miR-214 could target XBP1 expression via a yet unknown mechanism, suggesting that the miR-199a/miR-214 cluster may represent an example of miRs as both modulators and effectors of the ER stress response. Our present study has associated a new group of miRs with the ER stress response and demonstrated that at least some of them (e.g., miR-30b-5p and miR-30c-5p) may serve as modulators and effectors of the UPR to confer a survival advantage to the cell.

During tumorigenesis and tumor progression, the high rate of cancer cell proliferation demands elevated activities of ER protein folding, assembly, and transport. And cancer cells often experience nutrient deficit or hypoxia condition, which strongly induces accumulation of unfolded or misfolded proteins in the ER. All of these conditions can induce ER stress and activation of the UPR pathways^46^. Recent research in the cancer field has revealed that ER stress and the UPR are highly induced in various tumors and are closely associated with cancer cell survival and resistance to anti-cancer treatments^46^. Many of the PS-341-induced miRs, including miR-30b-5p, miR-30c-5p, miR-664–3p, miR-106–5p, miR-182–5p and miR-193–3p found by the present study to be upregulated in both HCC and breast cancer cell lines, have been previously suggested as onco-miRs during tumor progression. For instance, the miR-30s family has been demonstrated to play diverse functions in tumors. It was reported that miR-30a-5p suppresses tumor growth in colon cancer^47^. Ectopic expression of miR-30b/30d was found to promote the metastatic behavior of melanoma cells and reduce immune cell activation and recruitment^48^. In addition, miR-30e-3p is upregulated in human gliomas and ectopic miR-30e-3p overexpression promotes glioma cell invasiveness and angiogenesis^49^. Upregulation of miR-664 enhanced tumorigenesis and treatment with miR-664 siRNA reduced tumor growth, invasion, and metastasis in an orthotopic liver cancer model^50^. miR-106b was found to promote tumor cell proliferation in laryngeal carcinoma and exacerbate tumor invasion, metastasis and proliferation in breast cancer^51^,^52^. miR-106b expression is upregulated in higher stage tumors and correlated with tumor progression in breast cancer patients^53^. miR-182 was observed to be highly expressed in breast cancer cells, gliomas, melanoma and hepatocellular carcinoma, and to promote tumor metastasis, invasiveness and proliferation progression^54^,^55^,^56^. miR-193b was reported as a potentially novel prognostic marker in head and neck squamous cell carcinomas (HNSCC) that drives tumor progression, such as promotion of proliferation, migration, invasion, and tumor formation^57^. Consistent with these previous reports, our present study reveals that, miR-30b-5p, miR-30c-5p, miR-664–3p, miR-106–5p, miR-182–5p and miR-193–3p are induced by PS-341, demonstrates that at least miR-30b-5p and miR-30c-5p can serve as tumor facilitators which elevate cell viability and anti-apoptosis capacity, and shows that using the specific inhibitor of these miRs along with a proteasome inhibitor can lead to more effective apoptosis induction and better anti-tumor effects than using proteasome inhibitor alone. However, the mechanism by which PS-341 treatment upregulates these miRs remains unclear and is under our further investigation.

**Figure 6:**
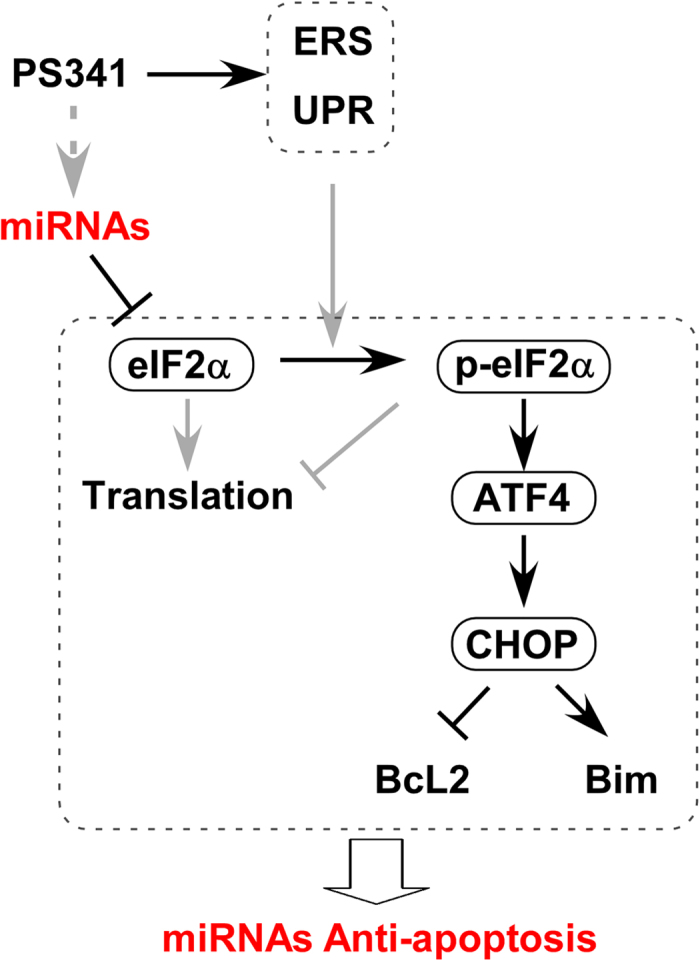
A proposed model for the PS-341-induced miRs to regulate the ER stress response and attenuate proteasome inhibition-mediated apoptosis in cancer cells via targeting eIF2α. Arrow, promote; flat arrow, suppress; dotted arrow, potential regulation.

Given the temporal relationship between the increase of p-eIF2α and the upregulation of miR-30b-5p and miR-30c-5p during ER stress, and considering the cell survival promoting property of these miRs, it is tempting to propose that the upregulated these miRs, via targeting and decreasing eIF2α, take the relay baton from p-eIF2α to continue decreasing protein flux into the ER for restoring ER proteostasis so that the cell can manage to survive from the ER stress irrespective of whether p-eIF2α is still in play (Fig. 6). Notably, unlike increasing p-eIF2α which increases ATF4 and can thereby lead to a CHOP-mediated pro-apoptotic pathway, these PS-341-induced miRs actually suppress the ATF4-CHOP-Bim signaling pathway. In terms of promoting cell survival, this difference represents to some extent an advantage of induction of these miRs over p-eIF2α elevation although both have the shared mission to inhibit general protein synthesis.

It should be pointed out that suppression of protein synthesis by proteasome inhibition has been well-documented but the underlying mechanism remains obscure. Since we have observed here that proteasome inhibitor PS341 treatment upregulates miR-30b-5p and miR-30c-5p in both normal and cancer cells and these miRs can target and decrease eIF2α, our findings also unveil a previously undocumented feedback mechanism by which impaired protein degradation suppresses general protein synthesis in the cell so that cellular proteostasis can be maintained at least temporarily.

## Conclusions

Our study reveals a novel miR-based mechanism by which the cell escapes from ER stress-induced cell death. Although the mechanism underlying the upregulation of these miRs during ER stress or proteasome inhibition has not been elucidated, the regulatory co-operation between ER stress and the miRs to promote cancer cell survival discovered here significantly improves our understanding of UPR mechanisms and of the maintenance of cancer cell proteostasis, which identifies a novel avenue to design more effective therapeutic strategies to battle cancer.

## Materials and Methods

### Materials

Reagents used in this study were obtained from the following sources: proteasome inhibitor PS341 (BD Biosciences, San Jose, CA); micrON® miR mimic Negative Control, micrOFF® miR inhibitor Negative Control, mimics and inhibitors for miR-30b-5p and miR-30c-5p, siR-Ribo™ Negative Control and si-eIF2α were purchased from RiboBio (RiboBio Co. Ltd, Guangzhou, Guangdong, China). miR-30b or miR-30c mimic, mimicker that can significantly improve the expression of mature miR-30b or 30c; miR-30b or miR-30c inhibitor, a LNA/OMe modified antisense oligonucleotide designed specifically to bind to and inhibit endogenous miR-30b or 30c molecule. Antibodies used in this study were purchased from following sources: anti-eIF2α, anti-p- eIF2α, anti-ATF4, anti-CHOP, anti-BcL2, anti-Bim, anti-GAPDH and horseradish peroxidase (HRP)-conjugated appropriate secondary antibodies (Cell Signaling, Danvers, MA, USA). Cell viability assay and cytotoxicity assay (Cell Counting Kit-8, CCK8 assay) kits were purchased from Sigma Corporation (Saint Louis, MO, USA). Propidium iodide (PI) and annexin V-FITC apoptosis detection kit were purchased from Keygen Company (Nanjing, China). Enhanced chemiluminescence (ECL) reagents were purchased from Santa Cruz Biotechnology Inc. (Santa Cruz). Lipofectamine™ RNAiMAX and Lipofectamine 2000 were purchased from Invitrogen Corporation (Carlsbad, CA, USA). pCMV-eIF2α-ORF plasmids were purchased from OriGene Technologies (Beijing, China).

### Cell culture and treatment

Both human hepatocellular carcinoma cells (HepG2) and breast cancer cells (MDA-MB-453), purchased from American Type Culture Collection (ATCC, Manassas, VA, USA), were grown in Dulbecco’s modified Eagle’s medium (DMEM, Invitrogen, Carlsbad, CA, USA) supplemented with 10% fetal bovine serum (FBS, Invitrogen), 100 U/mL benzyl penicillin and 100 U/mL of streptomycin, at 37 °C in a 5% CO_2_ atmosphere in a humidified incubator.

### Oligonucleotides, siRNA and transfection

The miR-30b-5p and miR-30c-5p mimic (or miRs mutant), inhibitor, and negative control (NC) for mimic (micrON® miR mimic Negative Control) and inhibitor (micrOFF® miR inhibitor Negative Control) respectively, were purchased from RiboBio (RiboBio Co. Ltd, Guangzhou, Guangdong, China). For depletion of eIF2α, three siRNAs were synthesized and purified by RiboBio (siRNAs sequences were: #1, GAAGGCGTATCCGTTCTAT; #2, GCTCCTCCTCGGTATGTAA; #3, GCAGGTTTGAATTGTTCTA;). Transfection of oligonucleotides, siRNAs or plasmids were performed using Lipofectamine 2000 (Invitrogen) as previously reported^49^, according to the manufacturer’s protocol. After transfection, cells were incubated at 37 °C for 24 hours until assessment.

### RNA extraction and real-time quantitative PCR (qRT-PCR)

Total RNA was isolated using trizol reagent (Invitrogen) according to the manufacturer’s instructions. The RNA concentration was measured using a NanoDrop ND-1000 spectrophotometer (NanoDrop Technologies Inc., Wilmington, Delaware, USA). The extracted RNA was pretreated with RNase-free DNase, and 500 ng of RNA from each sample were used for cDNA synthesis primed with the specific miR RT-primer purchased from RiboBio (RiboBio Co. Ltd, Guangzhou, Guangdong, China). For PCR amplification of cDNA, the primers of miRs and U6 were purchased from RiboBio, and the SYBR Premix Ex Taq Kit (Takara, Dalian, China) was used for qRT-PCR under the following conditions: 95 °C for 10 min, followed by 40 cycles of 95 °C for 15 s, 60 °C for 1 min. The expression of the miR was defined based on C_t_, and relative expression levels were calculated as 2^−[(Ct of miR)–(Ct of U6)]^after normalization with reference to the expression of small nuclear RNA U6.

### Western blot analysis

Cellular proteins were prepared in 1× sample buffer [62.5 mM Tris-HCl (pH 6.8), 10% glycerol, 2% SDS] and the concentrations were measured using Bio-Rad protein assay reagent (Bio-Rad Laboratories, Berkeley, CA, USA). An equal amount of total protein were fractionated by SDS-PAGE and electrically transferred onto polyvinylidene difluoride (PVDF) membranes (Millipore, Billerica, MA, USA). Rabbit or mouse primary antibodies (Cell Signaling), and appropriate horseradish peroxidase (HRP)-conjugated secondary antibodies (Cell Signaling) were used to detect the designated proteins. The bound secondary antibodies on the PVDF membrane were reacted with ECL detection reagents (Amersham Bioscience, Waltham, MA, USA).

### Luciferase assay

A full length of the human *eIF2α*-3′-UTR (3076 bp) containing miR-30b and miR-30c binding sites was cloned into vector pmiR-RB-Report^TM^ by RiboBio. 3 × 10^4^ cells/well were seeded in triplicate in 48-well plates and allowed to settle for 24 hours. 20 ng *eIF2α*-3′-UTR-luciferase reporter plasmid was transfected into cells using the Lipofectamine 2000 reagent (Invitrogen). Luciferase and control signals were determined 36 hours after transfection using a Dual Luciferase Reporter Assay Kit (Promega), according to manufacturer’s instruction. Three independent experiments were performed and the data were presented as the mean ± standard deviation (SD).

### Cell viability assay and cytotoxicity assay (Cell Counting Kit-8, CCK8 assay)

MDA-MB-453 and HepG2 cells were seeded in sextuplicate in a 96-well plate with 3000 cells per well, respectively, 1 day prior to transfection. The cells were transfected with different miR mimic, inhibitor or non-specific control (NC) in the following day. On the third day half of cells were treated with PS-341 (50 nM) for 24 h. Then the CCK8 assay was used to determine cell viability 48 hours after transfection. The absorbance at 450 nm was measured with a Quant Universal Microplate Spectrophotometer (BioTek, Winooski, VT, USA). Three independent experiments were performed and the data were presented as the mean ± SD.

### Annexin/PI double staining assay

A previously reported protocol was adopted with minor modifications^58^. Cells (48 h after transfection and 24 h after PS-341 treated) were washed twice with 1× phosphate buffered saline (PBS) and centrifuged. The cells were then re-suspended in ice-cold 1× binding buffer (500 μL). The cells suspensions with the binding buffer were moved into the 6-cm dish. Annexin V-FITC solution (4 μL) and PI (4 μL) were added to the cell suspensions and incubated for 30 minutes, in the dark. The apoptotic distribution of the cells in each sample was then determined using inverted fluorescence microscope. Three independent experiments were performed and the data were presented as the mean ± SD.

### Apoptosis analysis by flow cytometry

The cells (3 × 10^5^/well) were seeded in 6 cm-dish in antibiotic-free media, followed by transfection with mimics, inhibitors or control oligonucleotide using Lipofectamine 2000 (Invitrogen), and 24 h later followed by administration of PS-341 (50 nM). Cells were harvested by trypsinization, washed three times in PBS, and resuspended in 0.5 mL PBS. A fluorescein isothiocyanate (FITC)-conjugated monoclonal antibody specific for Annexin V and propidium iodide (PI) (Sigma) were added to the cells and incubated at 4 °C for 30 min. the cells stained by Annexin V and PI were then measured using flow cytometry (Becton Dickinson Biosciences, San Jose, CA, USA) and the data were analyzed by ModFit LT software package. The experiment was performed independently three times for each cell line.

### Statistical analysis

Two-tailed paired student’s *t* test was used to evaluate the statistical significance of the difference between two groups in all experiments. All data from three independent experiments were presented as mean ± SD. A *P* value < 0.05 was considered statistically significant.

## Additional Information

**How to cite this article**: Jiang, L. *et al.* A microRNA-mediated decrease in eukaryotic initiation factor 2α promotes cell survival during PS-341 treatment. *Sci. Rep.* **6**, 21565; doi: 10.1038/srep21565 (2016).

## Supplementary Material

Supplementary Information
