# Supplementary material for: A microRNA-mediated decrease in eukaryotic initiation factor 2α promotes cell survival during PS-341 treatment
Source: Sci Rep. 2016 Feb 22;6:21565. doi: 10.1038/srep21565 (PMC4761930; doi:10.1038/srep21565)
Supplement: Supplementary Information [file srep21565-s1.pdf]

**Supplementary information**

# A microRNA-mediated decrease in eukaryotic initiation factor 2α promotes cell survival during PS-341 treatment

**Lili Jiang, Dan Zang, Songgang Yi, Xiaofen Li, Changshan Yang, Xiaoxian Dong, Chong Zhao, Xiaoying Lan, Xin Chen, Shouting Liu, Ningning Liu, Hongbiao Huang, Xianping Shi, Xuejun Wang, and Jinbao Liu**

## Supplementary methods

### Microarray data processing and vitalization

HepG2 cells were treated with PS341 (50nM) or vehicle control for 12 hours. Then total miR from cultured cells was extracted using the mirVana miR Isolation Kit (Ambion, Waltham, MA, USA) according to the manufacturer's instructions. Microarray hybridization, data generation, and normalization were performed by the Kangchen Bio-tech (Shanghai, China) following standard Agilent protocols.

### Supplementary figures and legends

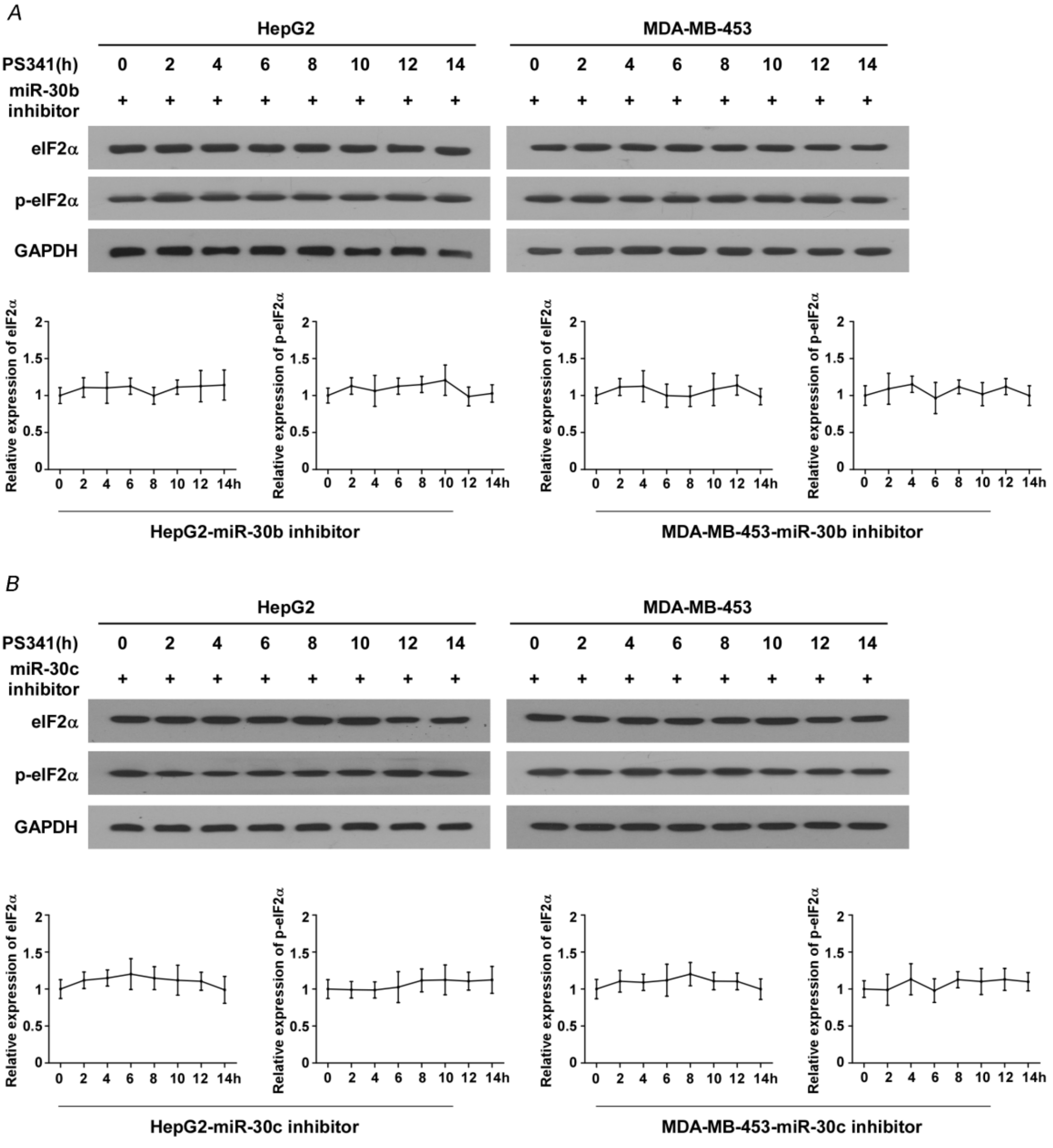

**Supplementary Figure 1. A time course comparison between eIF2α down-regulation, and eIF2α phosphorylation in the cells transfected with miRNA inhibitor after initiation of proteasome inhibition.** HepG2 and MDA-MB-453 cells transfected with miR-30b-5p **(A)** or miR-30c-5p **(B)** inhibitor, were harvested at the indicated duration after initiation of the treatment with proteasome inhibitor PS341 (50 nM). Western blot analyses of eIF2α and p-eIF2α in indicated cells were performed and shown. Lower panel, quantification of western blot analyses by Quantity One software. GAPDH was the loading control. Each bar represents the mean ± SD of three independent experiments; *$P$ < 0.05 vs. the NC of the same time point.

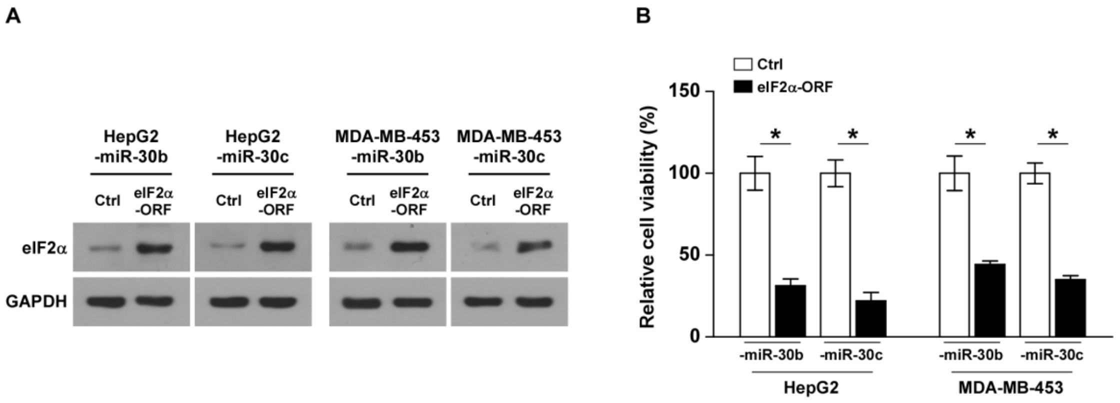


**Supplementary Figure 2. eIF2α-ORF rescues the pro-survival and anti-apoptotic action of miR-30b-5p and miR-30c-5p. (A)** The protein levels of eIF2α in the indicated cells that were transfected with pCMV-eIF2α-ORF which expresses a eIF2α (eIF2α-ORF) insensitive to the miRs, measured by western blot analysis. GAPDH served as loading control. (**B)** The cells viability of indicated cells analyzed by the CCK8 assay. Each bar represents the mean ± SD of three independent experiments; *$P$ < 0.05.
